# Born–Jordan Quantization and the Equivalence of the Schrödinger and Heisenberg Pictures

**DOI:** 10.1007/s10701-014-9831-z

**Published:** 2014-09-05

**Authors:** Maurice A. de Gosson

**Affiliations:** NuHAG, Faculty of Mathematics, University of Vienna, Oskar-Morgenstern-Platz 1, 1090 Vienna, Austria

**Keywords:** Heisenberg picture, Schrödinger picture, Quantization, Born and Jordan, Dequantization

## Abstract

The aim of the famous Born and Jordan 1925 paper was to put Heisenberg’s matrix mechanics on a firm mathematical basis. Born and Jordan showed that if one wants to ensure energy conservation in Heisenberg’s theory it is necessary and sufficient to quantize observables following a certain ordering rule. One apparently unnoticed consequence of this fact is that Schrödinger’s wave mechanics cannot be equivalent to Heisenberg’s more physically motivated matrix mechanics unless its observables are quantized using *this* rule, and *not* the more symmetric prescription proposed by Weyl in 1926, which has become the standard procedure in quantum mechanics. This observation confirms the superiority of Born–Jordan quantization, as already suggested by Kauffmann. We also show how to explicitly determine the Born–Jordan quantization of arbitrary classical variables, and discuss the conceptual advantages in using this quantization scheme. We finally suggest that it might be possible to determine the correct quantization scheme by using the results of weak measurement experiments.

## Introduction

In the Schrödinger picture of quantum mechanics (wave mechanics), the operators are constant (unless they are explicitly time-dependent), and the states evolve in time: $|\psi (t)\rangle =U(t,t_{0})|\psi (t_{0})\rangle$ where

1 $$\begin{aligned} U(t,t_{0})=e^{iH_{\mathcal {S}}(t-t_{0})/\hslash } \end{aligned}$$

is a family of unitary operators; the time evolution of $|\psi \rangle$ is thus governed by Schrödinger’s equation

2 $$\begin{aligned} i\hbar \frac{d\psi }{dt}=H_{\mathcal {S}}\psi ; \end{aligned}$$

$H_{\mathcal {S}}$ is an operator associated with the classical Hamiltonian function $H$ by some “quantization rule”. In the Heisenberg picture (matrix mechanics), the state vectors are time-independent operators that incorporate a dependency on time, while an observable $A_{\mathcal {S}}$ in the Schrödinger picture becomes a time-dependent operator $A_{\mathcal {H}}(t)$ in the Heisenberg picture; this time dependence satisfies the Heisenberg equation

3 $$\begin{aligned} i\hbar \frac{dA_{\mathcal {H}}}{dt}=i\hbar \frac{\partial A_{\mathcal {H}} }{\partial t}+[A_{\mathcal {H}},H_{\mathcal {H}}]. \end{aligned}$$

Schrödinger [22] (and, independently, Eckart [14]) attempted to prove shortly after the publication of Heisenberg’s result that wave mechanics and matrix mechanics were mathematically equivalent. Both proofs contained flaws, and one had to wait until von Neumann’s [24] seminal work for a rigorous proof of the equivalence of both theories (see the discussions in Madrid Casado [17] and Muller [19, 20]; both papers contain a wealth of historical details; also see van der Waerden’s [23] very interesting discussion Pauli’s unpublished letter regarding the (non)equivalence of wave mechanics and matrix mechanics). We will not bother with the technical shortcomings of Schrödinger’s and Eckart’s approaches here, but rather focus on one, perhaps more fundamental, aspect which seems to have been overlooked in the literature. We observe that it is possible to go from the Heisenberg picture to the Schrödinger picture (and back) using the following simple argument (see for instance Messiah [18] or Schiff [21]): a ket

4 $$\begin{aligned} |\psi _{\mathcal {S}}(t)\rangle =U(t,t_{0})|\psi _{\mathcal {S}}(t_{0} )\rangle \end{aligned}$$

in the Schrödinger picture becomes, in the Heisenberg picture, the constant ket

5 $$\begin{aligned} |\psi _{\mathcal {H}}\rangle =U(t,t_{0})^{*}|\psi _{\mathcal {S}}(t)\rangle =|\psi _{\mathcal {S}}(t_{0})\rangle \end{aligned}$$

whereas an observable $A_{\mathcal {S}}$ becomes

6 $$\begin{aligned} A_{\mathcal {H}}(t)=U(t,t_{0})^{*}A_{\mathcal {S}}U(t,t_{0}); \end{aligned}$$

in particular the Hamiltonian is

7 $$\begin{aligned} H_{\mathcal {H}}(t)=U(t,t_{0})^{*}H_{\mathcal {S}}U(t,t_{0}). \end{aligned}$$

Taking $t=t_{0}$ this relation implies that $H_{\mathcal {H}}(t_{0} )=H_{\mathcal {S}}$; now in the Heisenberg picture energy is constant, so the Hamiltonian operator $H_{\mathcal {H}}(t)$ must be a constant of the motion. It follows that $H_{\mathcal {H}}(t)=H_{\mathcal {S}}$ for all times $t$ and hence both operators $H_{\mathcal {H}}$ and $H_{\mathcal {S}}$ must be quantized using the *same* rules. A consequence of this property is that if we believe that Heisenberg’s “matrix mechanics” is correct and is equivalent to Schrödinger’s theory, then the Hamiltonian operator appearing in the Schrödinger equation (2) *must* be quantized using the Born–Jordan rule, and not, as is usual in quantum mechanics, the Weyl quantization rule.

### **Notation 1**

Real position and momentum variables are denoted $q,p$; more generally, for systems with $n$ degrees of freedom we write $q=(q_{1},..,q_{n})$, $p=(p_{1},\ldots ,p_{n})$. The boldface letters $\mathbf {q},\mathbf {p}$ are used to denote the corresponding quantum observables. Similarly, the quantum operator associated with a classical observable $A$ is denoted by $\mathbf {A}$ and we write $A\longleftrightarrow \mathbf {A}$. It is assumed throughout that this correspondence (“quantization”) is *linear*.

## The Born and Jordan Argument

We begin by shortly exposing the main arguments in Born and Jordan’s paper [2].

The paper of Born and Jordan was an attempt to put Heisenberg’s “magical paper” [15] on a firm basis (see Aitchison et al. [1] and van der Waerden [23] for interesting discussions of Heisenberg’s paper from a modern point of view). Following Heisenberg’s paper [15] Born and Jordan considered in [2] square infinite matrices

8 $$\begin{aligned} \mathbf {a}=(a(n,m))= \begin{pmatrix} a(00) &{}\quad a(01) &{}\quad a(02) &{}\quad \cdot \cdot \cdot \\ a(10) &{}\quad a(11) &{}\quad a(12) &{}\quad \cdot \cdot \cdot \\ a(20) &{}\quad a(21) &{}\quad a(22) &{}\quad \cdot \cdot \cdot \\ \cdot \cdot \cdot &{}\quad \cdot \cdot \cdot &{}\quad \cdot \cdot \cdot &{}\quad \cdot \cdot \cdot \end{pmatrix} \end{aligned}$$

where the $a(nm)$ are what they call “ordinary quantities”, *i.e.* scalars; we will call these infinite matrices (for which we always use boldface letters) *observables*. In particular Born and Jordan introduce momentum and position observables $\mathbf {p}$ and $\mathbf {q}$ and matrix functions $\mathbf {H}(\mathbf {p,q)}$ of these observables, which they call “Hamiltonians”. Following Heisenberg, they assume that the equations of motion for $\mathbf {p}$ and $\mathbf {q}$ are formally the same as in classical theory, namely

9 $$\begin{aligned} {\dot{\mathbf {q}}}=\frac{\partial \mathbf {H}}{\partial \mathbf {p}}, {\dot{\mathbf {p}}}=-\frac{\partial \mathbf {H}}{\partial \mathbf {q}}; \end{aligned}$$

limiting themselves deliberately to Hamiltonians which are polynomials in the observables $\mathbf {p}$, $\mathbf {q}$, that is linear combinations of monomials which are products of terms

10 $$\begin{aligned} \mathbf {H=p}^{s}\mathbf {q}^{r} \end{aligned}$$

they define the derivatives in (9) by the formulas, and show that the observables $\mathbf {p}$ and $\mathbf {q}$ satisfy the commutation relation

11 $$\begin{aligned} \mathbf {pq}-\mathbf {qp}=-i\hbar \mathbf {1} \end{aligned}$$

where $\mathbf {1}$ is the identity matrix; from this follows the more general identity

12 $$\begin{aligned} \mathbf {p}^{m}\mathbf {q}^{n}-\mathbf {q}^{n}\mathbf {p}^{m}=-i\hbar m\sum _{\ell =0}^{n-1}\mathbf {q}^{n-1-\ell }\mathbf {p}^{m-1}\mathbf {q}^{\ell }. \end{aligned}$$

Born and Jordan next proceed to derive the fundamental laws of quantum mechanics. In particular, pursuing their analogy with classical mechanics, they want to prove that energy is conserved; identifying the values of the Hamiltonian $\mathbf {H}$ with the energy of the system, they impose the condition ${\dot{\mathbf {H}}}=\mathbf {0}$ and show that this condition requires that

13 $$\begin{aligned} -i\hbar {\dot{\mathbf {q}}}=\mathbf {Hq-qH}\end{aligned}$$

14 $$\begin{aligned} -i\hbar \text {\ }{\dot{\mathbf {p}}}=\mathbf {Hp-pH.} \end{aligned}$$

Comparing with the Hamilton-like Eq. (9) this condition is in turn equivalent to

15 $$\begin{aligned} \mathbf {Hq-qH}=-i\hbar \frac{\partial \mathbf {H}}{\partial \mathbf {p}}\end{aligned}$$

16 $$\begin{aligned} \text { }\mathbf {Hp-pH}=i\hbar \frac{\partial \mathbf {H}}{\partial \mathbf {q}}. \end{aligned}$$

Now comes the crucial step. Given a classical Hamiltonian $H(p,q)=p^{s}q^{r}$ they ask how one should choose the observable $\mathbf {H}(\mathbf {p} ,\mathbf {q})$ so that these identities hold. Using the commutation formula (12) Born and Jordan show that the *only* possible choice is

17 $$\begin{aligned} \mathbf {H}(\mathbf {p},\mathbf {q})=\frac{1}{s+1}\sum _{\ell =0}^{s} \mathbf {p}^{s-\ell }\mathbf {q}^{r}\mathbf {p}^{\ell }. \end{aligned}$$

## Born–Jordan Quantization

Born and Jordan thus proved—rigorously—that the only way to quantize polynomials in a way consistent with Heisenberg’s ideas was to use the rule

18 $$\begin{aligned} p^{s}q^{r}\overset{\mathrm {BJ}}{\longrightarrow }\frac{1}{s+1}\sum _{\ell =0} ^{s}\mathbf {p}^{s-\ell }\mathbf {q}^{r}\mathbf {p}^{\ell }; \end{aligned}$$

equivalently, using the commutation relations (12):

19 $$\begin{aligned} p^{s}q^{r}\overset{\mathrm {BJ}}{\longrightarrow }\frac{1}{r+1}\sum _{j=0} ^{r}\mathbf {q}^{r-j}\mathbf {p}^{s}\mathbf {q}^{j}. \end{aligned}$$

In their subsequent publication [3] with Heisenberg they show that their constructions extend *mutatis mutandis* to systems with an arbitrary number of degrees of freedom. We will call this rule (and its extension to higher dimensions) the *Born–Jordan *(BJ)* quantization rule*. Weyl [25] proposed, independently, some time later (1926) another rule leading to the replacement of (18) with

20 $$\begin{aligned} p^{s}q^{r}\overset{\mathrm {Weyl}}{\longrightarrow }\frac{1}{2^{s}}\sum _{\ell =0}^{s} \begin{pmatrix} s\\ \ell \end{pmatrix} \mathbf {p}^{s-\ell }\mathbf {q}^{r}\mathbf {p}^{\ell }. \end{aligned}$$

It turns out that both rules coincide when $s+r\le 2$, but they are different as soon as $s\ge 2$ and $r\ge 2$.^1^ Both quantizations are thus not equivalent; as Kauffmann [16] observes, Weyl’s rule is the single most symmetrical operator ordering, whereas the BJ quantization is the equally weighted average of all the operator orderings.

These facts have the following consequence: if we insist that the Heisenberg and Schrödinger pictures be *equivalent*, then we *must* quantize the Hamiltonian in Schrödinger’s equation using BJ quantization. In fact, recall from formula (7) that the Heisenberg and Schrödinger Hamiltonians are related by

$$\begin{aligned} H_{\mathcal {H}}(t)=U(t,t_{0})^{*}H_{\mathcal {S}}U(t,t_{0}). \end{aligned}$$

Since $H_{\mathcal {H}}(t)$ is a constant of the motion we have $H_{\mathcal {H} }(t)=H_{\mathcal {H}}(t_{0})$ and hence $H_{\mathcal {H}}(t)=H_{\mathcal {S}}$ so the Heisenberg and Schrödinger Hamiltonians $H_{\mathcal {H}}(t)$ and $H_{\mathcal {H}}$ must be identical. But the condition $H_{\mathcal {H} }(t)=H_{\mathcal {H}}(t_{0})=H_{\mathcal {H}}$ means that $H_{\mathcal {H}}$ and hence $H_{\mathcal {S}}$ must be quantized using the Born and Jordan prescription.

An obvious consequence of these considerations is that if one uses in the Schrödinger picture the Weyl quantization rule (or any other quantization rule), we obtain two different renderings of quantum mechanics. This observation seems to be confirmed by Kauffmann’s [16] interesting discussion of the non-physicality of Weyl quantization.

## Generalization to Arbitrary Observables

We have been considering the quantization of polynomials for simplicity; in de Gosson and Luef [12] and de Gosson [6] we have shown in detail how to Born–Jordan quantize arbitrary functions of the position and momentum variables.

To find this general rule, we proceed as follows. Weyl quantization rule (20) can be viewed as a particular case of a very general rule, which we call the “$\tau$-rule”. Let us first consider a very simple example, that of the monomial $p^{2}q$ (for which both the BJ and the Weyl quantizations are identical^2^). We have

$$\begin{aligned} p^{2}q\overset{\mathrm {BJ}}{\longrightarrow }\frac{1}{3}(\mathbf {p} ^{2}\mathbf {q}+\mathbf {pqp}+\mathbf {qp}^{2}). \end{aligned}$$

Let now $\tau$ be an arbitrary real number and consider the following quantization rule

21 $$\begin{aligned} p^{2}q\overset{\tau }{\longrightarrow }(1-\tau )^{2}\mathbf {p}^{2}\mathbf {q} +2(1-\tau )\tau \mathbf {pqp}+\tau ^{2}\mathbf {qp}^{2} \end{aligned}$$

(it reduces to Weylo quantization if we choose $\tau =\frac{1}{2}$). If we integrate the right-hand side from $0$ to $1$ in $\tau$ we get

$$\begin{aligned} \int _{0}^{1}\left[ (1-\tau )^{2}\mathbf {p}^{2}\mathbf {q}+2(1-\tau )\tau \mathbf {pqp}+\tau ^{2}\mathbf {qp}^{2}\right] d\tau =\frac{1}{3} (\mathbf {p}^{2}\mathbf {q}+\mathbf {pqp}+\mathbf {qp}^{2}) \end{aligned}$$

which is precisely the BJ quantization of the monomial $p^{2}q$. More generally, we define the “$\tau$-quantization rule” for monomials by

22 $$\begin{aligned} p^{s}q^{r}\overset{\tau }{\longrightarrow }\frac{1}{2^{s}}\sum _{\ell =0}^{s} \begin{pmatrix} s\\ \ell \end{pmatrix} (1-\tau )^{\ell }\tau ^{s-\ell }\mathbf {p}^{s-\ell }\mathbf {q}^{r}\mathbf {p}^{\ell }. \end{aligned}$$

Again it reduces to the Weyl quantization when $\tau =\frac{1}{2}$; if we integrate the right-hand side from $0$ to $1$ in $\tau$ while observing that it follows from the properties of the beta function that

$$\begin{aligned} \int _{0}^{1}(1-\tau )^{\ell }\tau ^{s-\ell }d\tau =B(\ell +1,s-\ell )=\frac{\ell !(s-\ell )!}{(s+1)!} \end{aligned}$$

we recover the BJ quantization rule (18). This essential observation allows us to define the BJ quantization of an arbitrary classical observable. While we have done this from an operator-theoretical point of view in de Gosson [6] and de Gosson and Luef [12], we will follow here a more physical approach, along the lines of Kauffmann [16] with some modifications. We are working in $n$-dimensional configuration space, since it does not add any difficulty. The Weyl quantization $\mathbf {A}_{\mathrm {W}}(\mathbf {q},\mathbf {p})$ of a general observable $A(q,p)$ is unambiguously defined in its configuration space representation by the Fourier transform

23 $$\begin{aligned} \langle q_{2}|\mathbf {A}_{\mathrm {W}}|q_{1}\rangle =\left( \tfrac{1}{2\pi \hbar }\right) ^{n}\int e^{\frac{i}{\hbar }p(q_{2}-q_{1})}A(\tfrac{1}{2} (q_{1}+q_{2}),p)d^{n}p\mathbf {.} \end{aligned}$$

Define similarly $\tau$-quantization $A\overset{\tau }{\longrightarrow }\mathbf {A}_{\tau }$ in the configuration representation by

24 $$\begin{aligned} \langle q_{2}|\mathbf {A}_{\tau }|q_{1}\rangle =\left( \tfrac{1}{2\pi \hbar }\right) ^{n}\int e^{\frac{i}{\hbar }p(q_{2}-q_{1})}A(\tau q_{1}+(1-\tau )q_{2},p)d^{n}p\mathbf {;} \end{aligned}$$

of course $\mathbf {A}_{1/2}=\mathbf {A}_{\mathrm {W}}$. The BJ quantization $\mathbf {A}_{\mathrm {BJ}}$ is then defined as being the average of all the $\tau$-quantizations of $A(q,p)$ when the parameter $\tau$ goes from $0$ to $1$:

25 $$\begin{aligned} \mathbf {A}_{\mathrm {BJ}}=\int _{0}^{1}\mathbf {A}_{\tau }d\tau ; \end{aligned}$$

it follows from formula (24) that $\mathbf {A}_{\mathrm {BJ}}$ has the following configuration representation:

26 $$\begin{aligned} \langle q_{2}|\mathbf {A}_{\mathrm {BJ}}|q_{1}\rangle =\left( \tfrac{1}{2\pi \hbar }\right) ^{n}\int e^{\frac{i}{\hbar }p(q_{2}-q_{1})}\widetilde{A} (q_{1},q_{2},p)d^{n}p\end{aligned}$$

27 $$\begin{aligned} \text {with \ }\widetilde{A}(q_{1},q_{2},p)=\int _{0}^{1}A(\tau q_{1} +(1-\tau )q_{2},p)d\tau . \end{aligned}$$

The correspondence $A\overset{\mathrm {BJ}}{\longrightarrow }\mathbf {A} _{\mathrm {BJ}}$ thus defined reduces to the correspondence (18), (19) in the monomial case; it moreover has the property (shared with Weyl quantization) that to a real classical observable $A$ it associates a self-adjoint operator $\mathbf {A}_{\mathrm {BJ}}$ (this property, which is essential for any honest quantization theory, is not satisfied by the “$\tau$-quantization rule” $A\overset{\tau }{\longrightarrow }\mathbf {A}_{\tau }$, which is hence unphysical). It is easy to show using the formulas above that the BJ and Weyl quantization of Hamiltonians of the usual type “kinetic energy plus potential” are the same; we have shown in [6, 12] that BJ and Weyl quantization coincide for all Hamiltonians of the type

28 $$\begin{aligned} H=\sum _{j=1}^{n}\frac{1}{2m_{j}}(p_{j}-A_{j}(q,t))^{2}+U(q,t) \end{aligned}$$

where the vector and scalar potentials $A_{j}$ and $U$ depend on $q=(q_{1},...,q_{n})$ (and possibly on time $t$); this quantization is given by the usual formula

29 $$\begin{aligned} \mathbf {H}=\sum _{j=1}^{n}\frac{1}{2m_{j}}\left( -i\hbar \frac{\partial }{\partial x_{j}}-A_{j}(q,t)\right) ^{2}+U(q,t) \end{aligned}$$

(Messiah [18], Schiff [21]).

Let us briefly discuss in this context the property of canonical covariance. This property singles out Weyl quantization among all possible quantizations; it is probably thanks to this peculiarity that Weyl quantization superseded (at least among mathematical physicists) the BJ (and other possible quantization schemes). It is a very strong property (see the discussion at the end of the paper); it has allowed us to prove in [11] that Hamiltonian mechanics and quantum mechanics (when quantized using Weyl’s rule) are mathematically equivalent theories, *i.e.* that one can derive Schrödinger’s equation from Hamilton’s equations of motion, and vice versa. Canonical covariance means the following: let $$\mathrm{* }{Sp}(n)$$ be the symplectic group of the $$n$$-dimensional configuration space; it consists of all linear canonical transformations of the corresponding $$2n$$-dimensional phase space (we have given an elementary construction of $$\mathrm{* }{Sp}(n)$$ in de Gosson [8]). The elements of $$\mathrm{* }{Sp}(n)$$ are identified with $$2n\times 2n$$ matrices $$S$$ (“symplectic matrices”) satisfying the condition $$S^{T}JS=J$$ where the superscript $$T$$ indicates transposition and $$J= \begin{pmatrix} 0 &{}\quad I\\ -I &{}\quad 0 \end{pmatrix} $$ where $$0$$ and $$I$$ are the zero and identity $$n\times n$$ matrices. Now, to every symplectic matrix $$S$$ one can associate two unitary operators $$\pm \widehat{S}$$ acting on $$L^{2}(\mathbb {R}^{n})$$ (the square integrable functions); the set of all these operators form a group, the *metaplectic group* $$\mathrm{* }{Mp}(n)$$ (see de Gosson [4] for a detailed study of that group). The property of canonical covariance for a quantization rule $$A\longleftrightarrow \mathbf {A}$$ means that for every symplectic matrix $$S$$ we must have $$A\circ S\longleftrightarrow \widehat{S} \mathbf {A}\widehat{S}^{-1}$$ ($$A\circ S$$ is the new observable $$A\circ S(q,p)=A(S(q,p))$$). (Thus, a symplectic transformation of the coordinates in a classical observable corresponds at the operator level to conjugation by the corresponding metaplectic operator.) Now it is a mathematical theorem that there is only one quantization rule which enjoys this property: namely Weyl quantization. Therefore, if we use BJ quantization in place of Weyl quantization, we will lose canonical covariance for all observables which are not quantized the “Weyl way”. But this observation has no drastic consequences because, as we just mentioned, the Weyl and BJ quantizations of all physical Hamiltonians (28) are the same, and will thus have the property of canonical covariance. And there is another case where this remains true: formula (20) implies that monomials $$q_{j}^{2}$$, $$p_{j}^{2}$$, $$p_{j}q_{j}$$ (and, of course $$p_{j}q_{k})$$ have the same quantization in both schemes; it easily follows that the same is true for the generalized harmonic oscillator

30 $$\begin{aligned} H(p,q)=\sum _{j,k=1}^{n}a_{j}p_{j}^{2}+2b_{j}p_{j}q_{k}+c_{j}q_{j}^{2}. \end{aligned}$$

## Discussion

One might wonder at this point whether it is even at all possible to distinguish between these two quantization schemes. It follows from the discussion above that as far as ordinary Hamiltonians (28) or generalized oscillators (30) are concerned, we cannot. However, conceptually, there is an extremely important reason for which BJ quantization should be taken very seriously; it is related to the issue of *dequantization* (or “classicization”). Besides being canonically covariant, the Weyl rule has a very important, but rather unwelcome, property: it is one-to-one invertible because *every* continuous operator can be written uniquely as a Weyl operator (for a mathematical proof see e.g. de Gosson [4, 5]). This invertibility means that every quantum observable has a (unique) classical counterpart, and this is physically not tenable. The situation is very different when one uses BJ quantization. Let us explain this in some detail. We begin with the following observation, which is simple and subtle at the same time. Consider the BJ quantization $\mathbf {A}_{\mathrm {BJ} }\overset{\mathrm {BJ}}{\longleftrightarrow }A$ of some classical observable $A$. Born–Jordan operators are continuous operators, hence we can also view $\mathbf {A}_{\mathrm {BJ}}$ as a Weyl operator: $\mathbf {A}_{\mathrm {BJ} }=\mathbf {B}_{\mathrm {W}}\overset{\mathrm {W}}{\longleftrightarrow }B$ where $B$ is generally different from $A$. In de Gosson [6] and de Gosson and Luef [12] we have proven that the phase space Fourier transforms $\mathcal {F}A$ and $\mathcal {F}B$ of the classical observables $A$ and $B$ are related by the formula

31 $$\begin{aligned} \mathcal {F}B(q,p)=\Theta (q,p)\mathcal {F}A(q,p) \end{aligned}$$

where $\Theta$ is the real function given by

32 $$\begin{aligned} \Theta (q,p)=\frac{2\hbar }{pq}\sin \frac{pq}{2\hbar }. \end{aligned}$$

This formula implies that BJ quantization is neither one-to-one, nor invertible. In fact, every operator $\mathbf {A}$ has infinitely many precursors $A\overset{\mathrm {BJ}}{\longleftrightarrow }\mathbf {A} _{\mathrm {BJ}}$. Since quantization is linear, it is sufficient to verify this statement for $\mathbf {A}_{\mathrm {BJ}}=0$; writing again $\mathbf {A} _{\mathrm {BJ}}=\mathbf {B}_{\mathrm {W}}\overset{\mathrm {W}}{\longleftrightarrow }B$ we must have $B=0$ (and hence $\mathcal {F}B=0$) since the Weyl correspondence is one-to-one. In view of formula (31) this implies that $A$ is any observable such that

$$\begin{aligned} \left( \frac{2\hbar }{pq}\sin \frac{pq}{2\hbar }\right) \mathcal {F}A(q,p)=0. \end{aligned}$$

Since the function $\Theta (q,p)$ vanishes for all $(q,p)$ such that $pq=2N\pi \hbar$ ($N$ an integer $\ne 0$), this equality will hold for any classical observable whose Fourier transform vanishes outside the sets of phase space defined by these conditions; there is of course an infinite number of such choices. (See the interesting potential consequences for the limit $\hbar \rightarrow 0$ discussed by Kauffmann [16]). Similar considerations show that the correspondence $A\overset{\mathrm {BJ} }{\longleftrightarrow }\mathbf {A}_{\mathrm {BJ}}$ is in general not invertible. In fact, if it were, then to each Weyl operator $\mathbf {B}_{\mathrm {W}}$ would correspond a Born–Jordan operator $\mathbf {A}_{\mathrm {BJ}}$ such that $\mathbf {A}_{\mathrm {BJ}}=\mathbf {B}_{\mathrm {W}}$. The corresponding classical observable $A$ would then be determined by (31), but this is generally not possible because of the zeroes of the function $\Theta$.

It would certainly be interesting and useful to have explicit examples; the calculations are rather technical, and part of work in progress [13].

To conclude, if we believe in the equivalence of Heisenberg’s matrix mechanics and Schrödinger’s wave mechanics, then we must quantize both theories using the same correspondence. Matrix mechanics seems to be more physically motivated, being based on a natural notion, that of conservation of energy, which leads mathematically to the BJ quantization scheme, while there is no reason in Schrödinger’s theory to choose one particular quantization. This provides strong evidence that Born–Jordan quantization might very well be the right choice in quantum mechanics. Of course, to sustain this conjecture, it would be of primordial importance to test it experimentally. We suggest this could be done using weak measurements: as we have shown in [9], the notion of weak value can be expressed in two different ways, yielding different numerical results, depending on whether one uses Weyl or BJ quantization. Suppose in fact we have a classical observable $A$; we denote by $\mathbf {A}_{\mathrm {W}}$ and $\mathbf {A}_{\mathrm {BJ}}$ the corresponding Weyl and BJ quantizations. Let $|\psi \rangle$ be a pre-selected state and $|\phi \rangle$ a post-selected state; if these states are non-orthogonal the weak values of $\mathbf {A}_{\mathrm {W}}$ and $\mathbf {A}_{\mathrm {BJ}}$ with respect to the pair $(\phi ,\psi )$ are the complex numbers

$$\begin{aligned} \langle \mathbf {A}_{\mathrm {W}}\rangle _{\text {weak}}^{\phi ,\psi }=\frac{\langle \phi |\mathbf {A}_{\mathrm {W}}|\psi \rangle }{\langle \phi |\psi \rangle } , \ \langle \mathbf {A}_{\mathrm {BJ}}\rangle _{\text {weak}}^{\phi ,\psi }=\frac{\langle \phi |\mathbf {A}_{\mathrm {BJ}}|\psi \rangle }{\langle \phi |\psi \rangle }. \end{aligned}$$

In [10] we have shown that $\langle \mathbf {A}_{\mathrm {W}} \rangle _{\text {weak}}^{\phi ,\psi }$ can be calculated by averaging $A$ over the complex phase space function

$$\begin{aligned} \rho ^{\phi ,\psi }(q,p)=\frac{W(\phi ,\psi )(q,p)}{\langle \phi |\psi \rangle } \end{aligned}$$

where

$$\begin{aligned} W(\phi ,\psi )(q,p)=\left( \tfrac{1}{2\pi \hbar }\right) ^{n}\int e^{-\frac{i}{\hbar }pq^{\prime }}\phi (q+\tfrac{1}{2}q^{\prime })\psi ^{*}(q-\tfrac{1}{2}q^{\prime })d^{n}q^{\prime } \end{aligned}$$

is the cross-Wigner transform [4, 5], and in ([9]) that $\langle \mathbf {A}_{\mathrm {W}}\rangle _{\text {weak}}^{\phi ,\psi }$ is obtained similarly, but by averaging $A$ this time over

$$\begin{aligned} \rho _{\mathrm {BJ}}^{\phi ,\psi }(q,p)=\frac{W_{\mathrm {BJ}}(\phi ,\psi )(q,p)}{\langle \phi |\psi \rangle } \end{aligned}$$

where $W_{\mathrm {BJ}}(\phi ,\psi )$ is the modified cross-Wigner transform defined by (formula (46) in de Gosson [7])

$$\begin{aligned} W_{\mathrm {BJ}}(\phi ,\psi )=W(\phi ,\psi )*\mathcal {F}\Theta \end{aligned}$$

where $\mathcal {F}\Theta$ is the Fourier transform of the function (32).

## Footnotes

1 Ville Turunen, private communication.

2 We thank Maciej Blaszak for having pointed out this fact, thus correcting an error in an earlier draft.
